# Improvement of the Electrical-Mechanical Performance of Epoxy/Graphite Composites Based on the Effects of Particle Size and Curing Conditions

**DOI:** 10.3390/polym14030502

**Published:** 2022-01-27

**Authors:** Hendra Suherman, Radwan Dweiri, Abu Bakar Sulong, Mohd Yusuf Zakaria, Yovial Mahyoedin

**Affiliations:** 1 Department of Mechanical Engineering, Universitas Bung Hatta, Padang 25143, Indonesia; yovial@bunghatta.ac.id; 2 Department of Materials Engineering, Faculty of Engineering, Al-Balqa Applied University, Al-Salt 19117, Jordan; dr.dweiri@bau.edu.jo; 3 Centre for Materials Engineering and Smart Manufacturing, Faculty of Engineering and Built Environment, Universiti Kebangsaan Malaysia, Bangi 43600, Malaysia; abubakar@ukm.my (A.B.S.); zakaria@ukm.my (M.Y.Z.)

**Keywords:** epoxy/G composites, electrical conductivity, tensile strength, curing process, thermal stability

## Abstract

This study aims to improve the electrical-mechanical performance of traditional epoxy/graphite composites for engineering applications. The improvement in the properties of these composites depended on the incorporation of different sizes of graphite particles of the same type and controlling their curing process conditions. The thermal properties and microstructural changes were also characterized. A maximum in-plane electrical conductivity value of approximately 23 S/cm was reported for composites containing 80 wt.% G with a particle size of 150 µm. The effect of combining large and small G particles increased this value to approximately 32 S/cm by replacing the large particle size with 10 wt.% smaller particles (75 µm). A further increase in the electrical conductivity to approximately 50 S/cm was achieved due to the increase in curing temperature and time. Increasing the curing temperature or time also had a crucial role in improving the tensile strength of the composites and a tensile strength of ~19 MPa was reported using a system of multiple filler particle sizes processed at the highest curing temperature and time compared to ~9 MPa for epoxy/G150 at 80 wt.%. TGA analysis showed that the composites are thermally stable, and stability was improved by the addition of filler to the resin. A slight difference in the degraded weights and the glass transition temperatures between composites of different multiple filler particle sizes was also observed from the TGA and DSC results.

## 1. Introduction

Researchers are still making efforts to achieve better electrical-mechanical performance of conductive polymer composite materials (CPCs) for various engineering applications [1,2,3]. Other properties of these composites that are also of interest are their thermal conductivity, thermal stability, corrosion resistance, and the lightweight nature of the composites [4,5,6]. Improvement of the electrical properties of CPCs can be achieved by using conductive fillers such as graphite (G), carbon black (CB), carbon nanotubes (CNTs), graphene nanoplatelets (GNPs), and carbon fibers (CF) [7,8,9,10,11]. Different factors play crucial roles in determining the final properties of CPCs: the filler content, size, shape, and distribution in the matrix have been shown to be the most important [11,12,13,14]. If a conductive filler with a specified particle shape and size is used, then optimizing the processing technique for the best distribution of the filler particles in the matrix is crucial. Successfully achieving uniform distribution of the filler in the matrix results in better mechanical and electrical properties and lower filler loading, which in turn reduces the weight and cost of the product. Researchers have typically used high conductive filler loadings of up to 90 wt.% to achieve high electrical conductivity in engineering applications, such as for the bipolar plate in polymer electrolyte membrane fuel cells (PEMFCs), for which the US Department of Energy (DOE) set a target electrical conductivity of greater than 100 S/cm [15]. Many studies have been conducted in response to achieve the DOE target [16,17]. However, a degradation of the mechanical properties was obvious in the composite material with a high filler content, and its fabrication process was also more difficult [18]. However, the diversity in material types and their processing techniques and the operator’s skills still open the door to running increasingly fundamental studies to understand the overlap between the structure-property-processing relationship and the performance of CPCs. To date, improved electrical-mechanical properties at lower filler content have only been achieved by using multifiller conductive composites with micro- and nano-sized particles and fibers.

Among the various conductive fillers used in CPCs, graphite is still of interest because it has high electrical conductivity and various shapes and sizes, and it is easily obtained using conventional processes [19,20]. The synergistic effect of compositing small particle size and large particles in the polymer matrix has been extensively investigated by many researchers [21,22]. The use of large particles as a main filling component in the matrix or vice versa was also studied. Researchers found that combining a larger primary conductive filler with a smaller secondary conductive filler could increase the electrical conductivity value and improve the mechanical properties of the conductive composite. [23,24,25]. Chunhui et al. [26] studied the influence of the incorporation of different sizes of conductive filler materials on the electrical conductivity of composite materials for the applications of bipolar plates in fuel cells. They found that the electrical conductivity of the produced composite material was also influenced by the incorporation of multiple conductive filler materials, and thus, the appropriate particle size of the conductive filler increased the density of the composite material and reduced the electrical resistivity. Hui et al. [16] used different graphite particle sizes in their study and found that the electrical conductivity increased with increasing graphite particle size. The electrical properties of composites containing expanded graphite (EG) of different sizes (30, 50, 150, and 300 µm) and phenolic resin as a matrix were investigated by Dhakate et al. [27], and they found that the highest electrical conductivity was obtained in EG/phenolic composites with the largest EG size of 300 µm. The effect of particle size and graphite morphology on the electrical conductivity of graphite/polypropylene (G/PP) composite bipolar plates was studied by Derieth et al. [28]. The load of conductive filler material was maintained at 78 wt.%, and compounds with three different sizes of flake-shaped conductive fillers with sizes of 5, 10, and 20 µm were produced. The results showed that the electrical conductivity of G/PP composites increased with the decreasing particle size of flake-shaped graphite and that the flake-shaped particles were able to produce G/PP composites with higher electrical conductivity than those containing sphere-shaped graphite. Suherman et al. [29] investigated the effect of hybrid conductive filler loading up to 50 wt.% on the electrical conductivity and tensile strength of conductive polymer composite materials. As the main filler, 74 µm graphite particles were used, and 13 and 5 µm G particles were used as the secondary conductive fillers. They found that the incorporation of a smaller conductive filler resulted in a significant increase in electrical conductivity and tensile strength compared to CPCs using only the main conductive filler with a size of only 74 µm.

On the other hand, many studies have highlighted the optimization of the processing parameters of fabrication techniques to improve the electrical and mechanical performance of CPCs [24,30]. The improvement of the properties is associated with the dispersion of the filler in the polymers, which depends on the adopted processing method [31,32]. A study on the composites polyethersulfone (PES) filled with G and CB prepared by solution blending technique reported an electrical conductivity four times higher than that prepared by powder mixing method [33]. Hu et al. [34] conducted a study on the effect of curing and mixing processes on the electrical conductivity of epoxy multiwall carbon nanotube (MWCNT) composites. They found that the mixing conditions and curing temperature were the controlling factors for increasing the electrical conductivity of the resulting composite material. Chandrasekaran et al. [35] studied the mechanical-thermal-electrical performance of epoxy/GNPs processed by two dispersion methods: three-roll milling and sonication combined with high speed shear mixing technique. Composites prepared by three-roll milling showed higher electrical conductivity of almost three orders compared to that prepared by the sonication process, which was related to the dispersion degree and the spatial distribution of filler. Li et al. [36] analyzed the influence of post-curing temperature and preparation method on the mechanical, electrical, and physical properties of G/CF/copper/phenolic resin composites. It was found that the properties of composites improved with increasing post-curing temperature.

Finally, most of the previous studies in the literature focused on the improvement of the electrical and mechanical properties of polymer/G composites either by using a specified size of G particles incorporated into the composite [37,38,39,40,41] or by hybridizing large G particles with other types of smaller conductive fillers such as CB, CNTs, and CF [42,43,44,45,46]. Few studies investigated the synergistic effect of using different sizes of the same kind of conductive filler in the composite [47]. Mohd Radzuan et al. [47] fabricated a PP/G composite with a 75 wt.% G content and found that the combination of two G sizes of 40 and 200 µm exhibited better electrical conductivity and maintained good flexural strength. So, the motivation behind this study was the need to further understand the effectiveness of combining large G particles with smaller G particles of the same kind in epoxy/G composite and to determine to which degree the in-plane electrical conductivity and the tensile strength could be improved. The epoxy resins were usually used due to their excellent adhesion to other materials, high mechanical strength and stiffness, and high thermal and chemical resistance, which meet the requirements for different types of composites and applications. Szeluga et al. [48] described, in his review on epoxy composites, some recent works on the effect of combinations of graphene particles with other fillers on the mechanical, electrical, and thermal properties. The other issue of interest in this study was the effect of processing conditions on the composite properties. A compromise between the electrical and mechanical properties of conductive polymer composites is still a target for many researchers. Other properties, such as the thermal stability of the composites and the resulting microstructural changes, were also investigated. Filler loading of 65 to 80 wt% was used in this study due to the low value of in-plane electrical conductivity obtained below this percentage range. It is worth to mention that the work carried out in this study represents an extension to a previous work done by the same author, Suherman et al. [23], on epoxy/G composites, who reported a maximum in-plane electrical conductivity of about 28 S/cm and a maximum tensile strength of about 14 MPa by optimizing the curing conditions at 80 wt.% G content of 150 µm particle size (G150).

## 2. Materials and Methods

### 2.1. Materials

**Figure 1:**
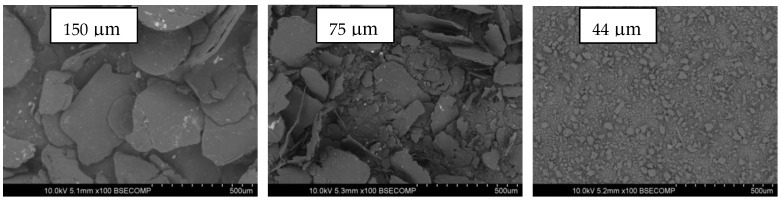
Graphite powders with different particle sizes.

In this study, three different sizes of the same graphite particle type were used as conductive fillers: particles with an average size of 150 µm (designated G150) were used as the main conductive filler up to 80 wt.%, and smaller average particle sizes of 75 µm (G75) and 44 µm (G44) were used as secondary fillers up to 10 wt.%. Graphite powder with a density of 2.19 g/cm^3^ was purchased from Asbury Carbon, New Jersey. The binder (matrix) used was epoxy resin (1.1 g/cm^3^ density) and hardener (635 thin epoxy resin) of 1.04 g/cm^3^ density with a 4:1 ratio fast epoxy hardener, which had a low viscosity of 6 poise and was supplied by a US composites company. The SEM images of the different G particle sizes are shown in Figure 1. The G particles presented irregular plate-like shapes with slightly rough surfaces, and the particles were diverse in size around the average value for each type.

### 2.2. Preparation of Epoxy/G Composites

The epoxy/G composites were prepared as follows: (i) the epoxy resin and the hardener were initially mixed with a composition ratio of 4:1 according to the manufacturer datasheet, using a mechanical stirrer (IKA-RW-20 digital, IKA-Werke GmbH & Co. KG, Staufan im Breisgau, Germany) at a rotation speed of 250 rpm for 10 min; (ii) the G conductive particles were then added to the epoxy-hardener mixture during stirring based on the predetermined compositions shown in Table 1; and (iii) the epoxy/G mixture was poured into a (10 × 10) cm^2^ square mold and placed in an oven (TE0-11, PT. Indotara Persada, Jakarta, Indonesia). The process of curing the resin was carried out at different curing temperatures (110, 130, and 150 °C) and different curing times (60, 90, and 120 min) to obtain the conductive polymer composite materials. Twelve different formulations of epoxy/G composites are shown in Table 1.

### 2.3. Characterization

The in-plane electrical conductivity of the composites was measured according to the ASTM C611 method using the conventional four-probe technique at a constant current supply [2]. The tensile strength of the composites was measured according to the ASTM D3039 standard using a universal testing machine (Wew 300C, TIME Group Inc, Tianjin China). Thermogravimetric analysis (TGA) and differential scanning calorimetry (DSC) were carried out using a Mettler Toledo brand machine (SDTA 851e type, Greinfensee, Switzerland) in the temperature range of 30 to 600 °C at a heating rate of 20 °C/min in a nitrogen environment. Scanning electron microscopy (Hitachi Type S-3400 N, Hitachi High–Tech, Kawasaki, Japan) was used for microstructural observations of gold-coated samples.

## 3. Results and Discussion

### 3.1. In-Plane Electrical Conductivity Results

**Figure 2:**
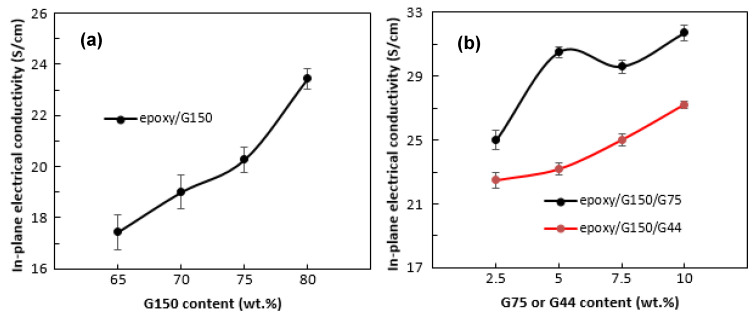
In-plane electrical conductivity values of the epoxy/G composites with different G particle sizes and contents: (**a**) epoxy/G150 and (**b**) epoxy/G150/G75 and epoxy/G150/G44 prepared at 110 °C for 60 min.

**Figure 3:**
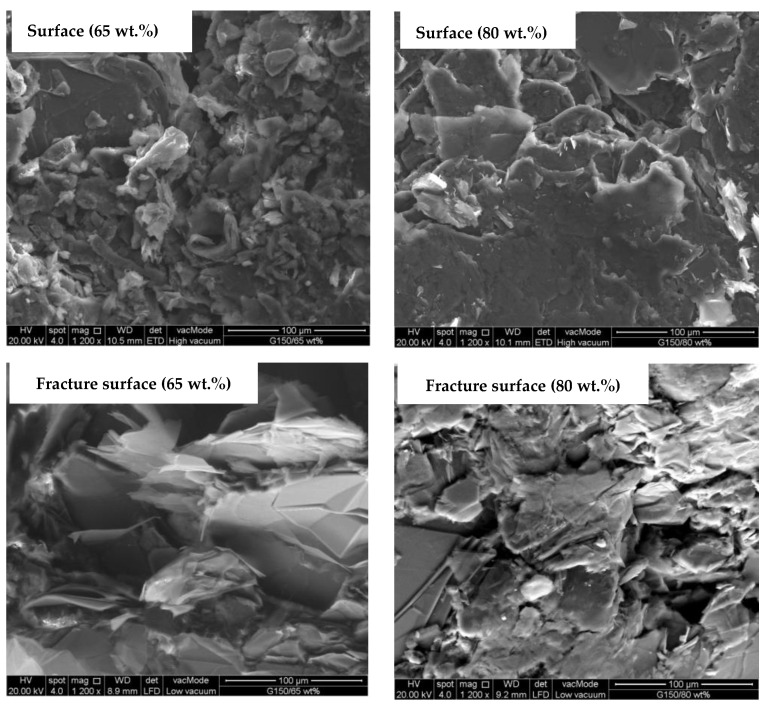
SEM surface and fracture surface images of epoxy/G150 at 65 and 80 wt.% filling loads of G prepared at 110 °C for 60 min.

The values of the in-plane electrical conductivity of the epoxy/G composites with different G filler loadings at different types of G particle sizes are shown in Figure 2. The composites in Figure 2 were prepared at curing conditions of 110 °C and 60 min. As shown in Figure 2a, the in-plane conductivity increases with the addition of 65 to 80 wt.% G filler (G150) to the epoxy/G composites. This phenomenon indicated that the in-plane electrical conductivity value obtained is in accordance with the percolation theory, i.e., the electrical conductivity increases with increasing conductive filler load in the polymer matrix [49,50]. An increase in the in-plane electrical conductivity with filler loading was expected and reported by many researchers [26,27,28,29], and the higher the filler loading was, the more network paths there were for electrons to pass through. A maximum value of about 23 S/cm was achieved for epoxy/G150 composites at 80 wt.% G150. The comparison of these results with those obtained in previous studies is dependent on the type and size of G and matrix used as well as the processing techniques. A study conducted by Suherman et al. [13] on the epoxy/G composites reported a value of only about 7 S/cm in-plane conductivity at 80 wt.% of 44 µm G particle size. In a similar work by Suherman et al. [23], by optimizing the curing conditions, the conductivity value reached about 28 S/cm for epoxy/G composites using 150 µm G particle size. Dweiri and Sahari [18], who prepared PP/G composite materials by melt compounding of the components and then compression molding the compounds, reported a 7 S/cm in-plane electrical conductivity for composites containing 80 wt.% G of 10 µm particle size, while the value reached up to 23 S/cm for the composites prepared by using a solution blending technique due to the better dispersion of the fillers. Another significant factor that affects the value of electrical conductivity, as mentioned earlier, is dispersion of the conductive filler in the polymer matrix [15,51]. The agglomeration and uneven distribution of the conductive fillers results in a low electrical conductivity of the composite material, which was clearly observed from the SEM images of the surface and fracture surface of the epoxy/G150 composites in Figure 3. The SEM surface images showed that the large G particles with non-regular plate-like shapes distributed on the matrix sometimes oriented and folded over each other while the smaller particles agglomerated on the in-plane surface of the large particles, leaving unfilled voids between the large particles in the through-plane direction. The lack of a binder in some areas and the less particle-to-particle contacts in the in-plane surface of the composite material resulted in less continuity in the phase of the composite material. The continuity in the phase appeared much better when the plate-like particles oriented, which positively reflected on the value of the in-plane electrical conductivity, especially at 80 wt.% G. The smooth surfaces of the particles and the interfacial cracks indicated poor adhesion between the matrix and the particles, as well as brittle-like behavior, as shown from the fracture surfaces of the epoxy/G150 composites. Even though the increase in the value was approximately 34% at 80 wt.% G150 compared to that at 65 wt.%, the value of 23 S/cm is still not high enough and needs to be improved to meet the requirements for engineering applications.

**Figure 4:**
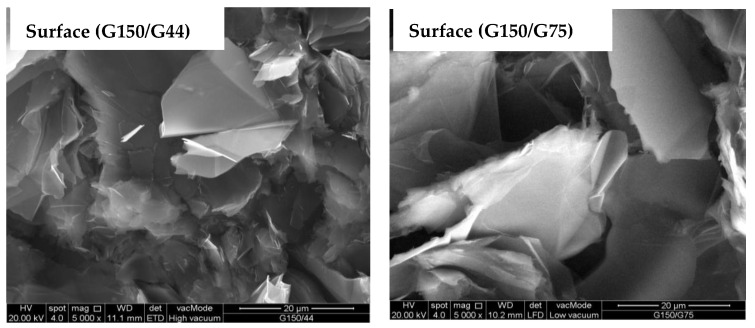

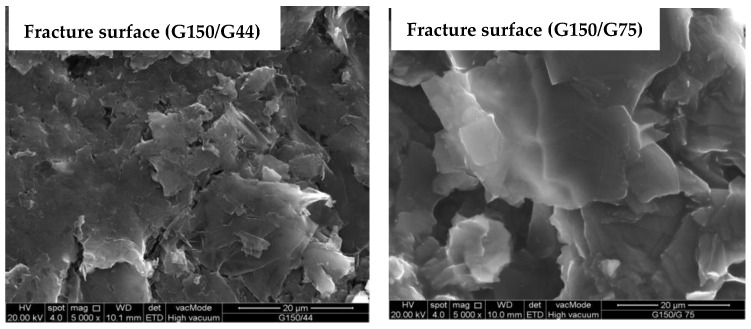
SEM surface and fracture surface images of epoxy/G150/G75 and epoxy/G150/G44 at 80 wt.% overall filling load prepared at 150 °C for 120 min.

Epoxy/G150 with 80 wt.% G150 was selected as the master batch formula based on its high in-plane electrical conductivity value. In an attempt to further improve the in-plane electrical conductivity and to determine the effect of the incorporation of different sizes of G particles into the matrix, up to 10 wt.% of G150 was replaced by smaller G particle size, G75 or G44, and the results are shown in Figure 2b. A clear improvement in the electrical conductivity was observed by the addition of different G sizes. An increase of approximately 35% in the electrical conductivity compared to that of the master batch epoxy/80 wt.% G150 sample was achieved by the combination of 70 wt.% of the largest size G150 with 10 wt.% of a smaller size G75. The combination of G150 with a smaller 10 wt.% G44 size resulted in an approximately 16% improvement in the electrical conductivity. Chunhui et al. [26] studied the effect of combining different sizes of conductive fillers on the electrical conductivity values for bipolar plate composite materials. They found that the electrical conductivity increased by 37% when combining 10 wt.% of the small particles (<45 µm) with the larger particles (>90 µm) at an overall G loading of 60 wt.% and attributed that to the reduction in the pore volume due to the accumulation of the large G size and the increase in the particle-to-particle contacts. The conductivity decreased when the proportion of small G size increased. Mathur et al. [52] also reported an increase in the electrical conductivity of epoxy/G composites when incorporating up to 15 wt.% synthetic graphite (SG) with a particle size <1 µm into the natural graphite (NG) of particle size (75–150 µm) and attributed that to the ability of the small particles to fill the voids between NG flakes. The SEM images of the surfaces and fracture surfaces of epoxy/G150/G44 and epoxy/G150/G75 composites in Figure 4 showed a similar microstructure as the epoxy/G150 composites. The smaller G particle sizes (G44) might have a higher tendency to agglomerate and unevenly distribute and did not sufficiently fill the micro-voids that existed in the composite, which in turn did not contribute to increasing the electrical conductivity compared to that of the larger G particles (G75). Hui et al. [16] found that the increase of the particle size in epoxy/G composites decreased the number of particles and the contact resistance, which consequently improved the conductivity. Chunhui et al. [26] investigated the particle size gradation of different conductive filler particle sizes in the polymer composite and reported that the optimal small particle size (d), which can enter into the accumulation of large particle sizes (D), is d = 0.154D in the closest accumulation mode and d = 0.414D in the loosest accumulation mode. Based on these ratios, the optimal small particle size when using large G particle sizes of G150 is d = ~23 µm for the closest accumulation and d = ~62 µm for the loosest accumulation, which deviated from the smaller particle sizes used in this study (i.e., G44 and G75).

**Figure 5:**
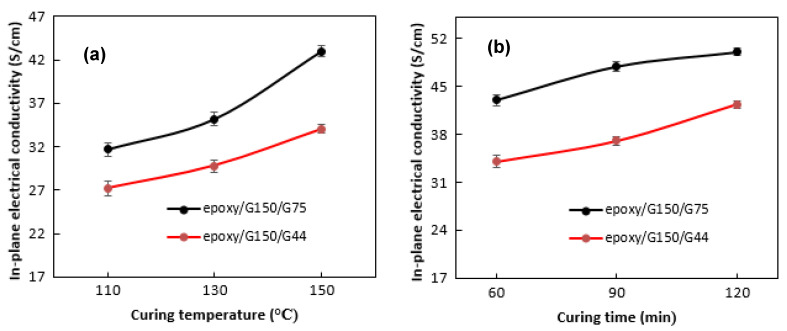
The in-plane electrical conductivity values of (**a**) epoxy/70 wt.% G150/10 wt.% (G44 or G75) prepared at 60 min and different curing temperatures; (**b**) epoxy/70 wt.% G150/10 wt.% (G44 or G75) prepared at 150 °C and different curing times.

The curing conditions were found to have a crucial role in determining the in-plane electrical conductivity, as shown in Figure 5. At a specified curing time of 60 min, the conductivity of the epoxy/G150/G75 composite was increased sharply from ~32 to 43 S/cm by increasing the curing temperature from 110 to 150 °C (Figure 5a). Hui et al. [16] studied the effect of curing temperature on the electrical conductivity of novolac epoxy/G composite and reported an increase of about 10% when raising the temperature. Suherman et al. [13] in a previous work found the same trend when producing epoxy/40 wt.% G/5 wt.% CNTs composites and achieved an increase of about 40% (from 14.5 to 21 S/cm) in the electrical conductivity by increasing the curing temperature from 80 to 120 °C. They attributed the increase in the electrical conductivity to the matrix viscosity, which decreased at higher curing temperatures, allowing the G filler to be more evenly dispersed, and thus a conductive network can be formed more easily in the matrix. The increase in the conductivity was less pronounced for the epoxy/G150/G44 composite (from ~27 S/cm to 34 S/cm). Hu et al. [34] and Martin et al. [53] found that the processing temperature affected the viscosity and electrical conductivity of the polymer matrix in epoxy/CNTs composites. The electrical conductivity properties were affected by the ability of the polymer matrix to wet the conductive filler. The higher the temperature of the forming process, the lower the bonding of the polymer composite material mixture. This condition increases the movement of the conductive filler in the polymer matrix progressively with increasing processing temperature. The same trend was also observed in Figure 5b when increasing the curing time from 60 to 120 min at a specified curing temperature of 150 °C, and a further increase in the conductivity was also achieved, which reached up to 50 S/cm for the epoxy/G150/G75 composite and up to 42 S/cm for the epoxy/G150/G44 composite. This was because the formation of conductive tissue in the matrix is strongly influenced by the curing time used [16,34].

### 3.2. Tensile Strength Results

**Figure 6:**
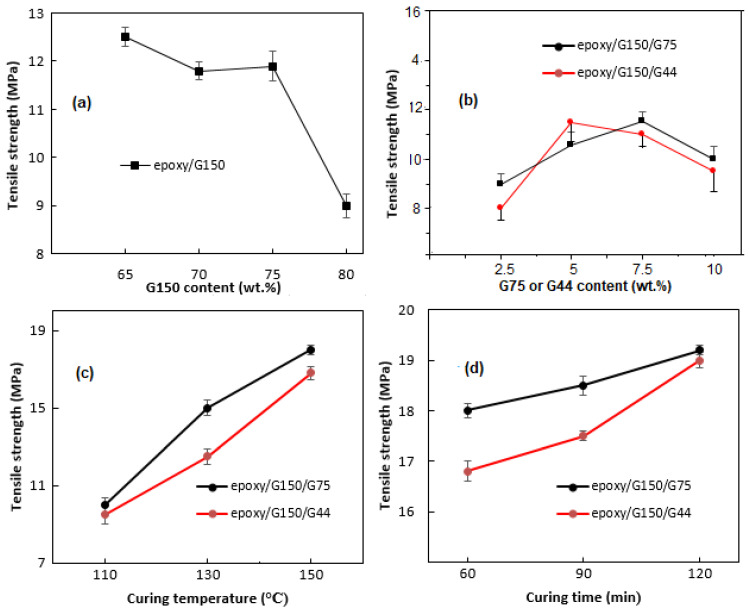
The tensile strength values of epoxy/G composites: (**a**) epoxy/G150 prepared at 110 °C, 60 min; (**b**) epoxy/70% G150/G75 and epoxy/70% G150/G44 prepared at 110 °C, 60 min; (**c**) epoxy/70 wt.% G150/10 wt.% (G44 or G75) prepared at 60 min and different curing temperatures; (**d**) epoxy/70 wt.% G150/10 wt.% (G44 or G75) prepared at 150 °C and different curing times.

The results of the tensile test for all types of composites are shown in Figure 6. In general, the tensile strength decreased with the addition of the filler, as shown in Figure 6a, and the lowest value was reported at 80 wt.% G150. The decrease in the strength by increasing the G content was expected, as the amount of the resin decreased and the porosity increased [16,52]. Poor adhesion between the binder and the filler was usually the reason for the degradation of the mechanical properties, which was evident from the microstructural observations shown in Figure 3 and Figure 4, represented by the existence of interfacial cracks, voids, and clear filler surfaces. Compared to the tensile strength of 9 MPa for epoxy/G150 at 80 wt.% G content, the tensile strength increased slightly to ~10 MPa by combining 70 wt.% G150 with 10 wt.% G75 and to ~9.5 MPa in case of 10 wt.% G44, as shown in Figure 6b. Increasing the curing temperature and the curing time had a positive impact on the tensile strength values of the composites, as shown in Figure 6c,d. The highest tensile strength was obtained at the highest curing temperature of 150 °C. The test results showed a tensile strength of ~19 MPa for epoxy/G150/G75 and G150/G44/epoxy composites. This is due to the stronger adhesion formed between the conductive filler material and the resin, which is attributed to the increase in the degree of cross linking [16,23]. The increase in tensile strength was slightly more pronounced for epoxy/G150/G44 composites by increasing the curing time from 60 to 120 min, while a slight improvement occurred in the case of epoxy/G150/G75 composites. Chunhui et al. [26] in their study on aluminate cement/graphite and sodium silicate cement/graphite found that as the weight ratio of small size G particles (<45 µm) to big size G particles (>90 µm) increased, the strength of the composites increased, and it was higher than that of the composites using one type of the same-sized graphite. Suherman et al. [23], in a previous study, investigated the effect of curing conditions on epoxy/G composites with 150 µm G particles (G150), and found that the tensile strength value fluctuated in the range 10 to 14 MPa at 80 wt.% G150 with the changes in curing temperature and time, and no clear trend was noticed.

### 3.3. Thermal Properties of the Composites

**Figure 7:**
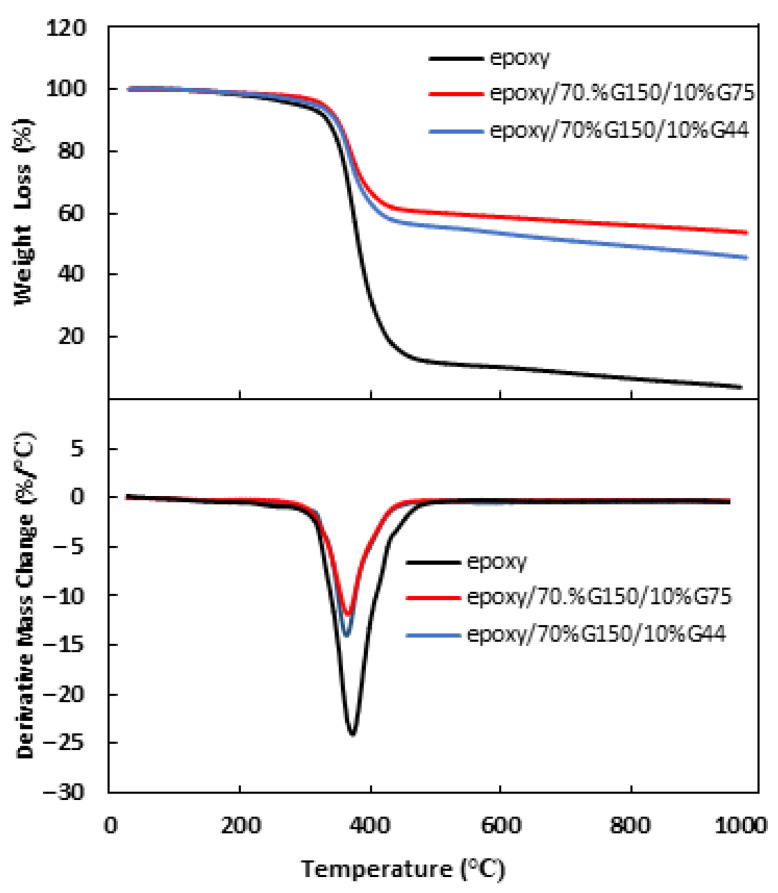
TGA and DTG curves of pure epoxy, epoxy/G150/G44 and epoxy/G150/G75 composites at 70 wt.% G150 and 10 wt.% G44 or 10 wt.% G75 prepared at 150 °C for 120 min.

**Figure 8:**
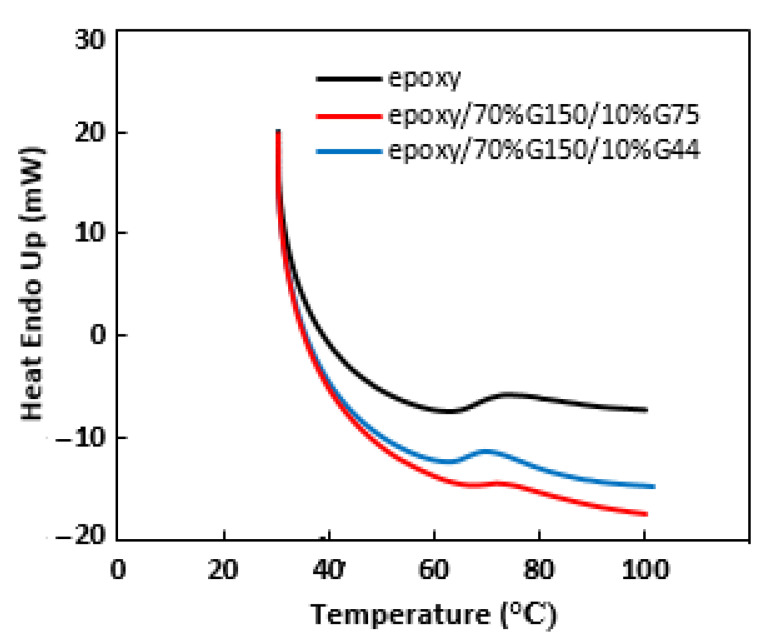
DSC curves of pure epoxy, epoxy/G150/G44 and epoxy/G150/G75 composites at 70 wt.% G150 and 10 wt.% G44 or 10 wt.% G75 prepared at 150 °C for 120 min.

The thermal stability of pure epoxy and epoxy/70 wt.% G150/10 wt.% (G44 or G75) composites was investigated, and the TGA and DTG curves are shown in Figure 7. Maximum decomposition temperatures and weight losses of the epoxy and epoxy/G composites at different temperatures are tabulated in Table 2. The samples are almost thermally stable, with minor weight loss percentages occurring up to 200 °C, reaching approximately 0.24% and 0.33% for epoxy/G150/G75 and epoxy/G150/G44 composites, respectively, at an overall filling load of 80 wt.%. This minor weight loss might be attributed to the evolution of moisture and volatiles [54]. The weight loss percentages at the maximum decomposition temperatures were 11.84% for epoxy/G150/G75 and 14.01% for epoxy/G150/G44 composites. This might be attributed to the degradation of epoxy itself [54]. Hui et al. [16] also reported a 0.6% weight loss of the novalac epoxy/NG composite material at a temperature of 100 °C. As expected, the degraded weight decreased by filling the epoxy with G particles [55]. Bhagat [56] attributed the improvement of thermal stability of epoxy/G composites at 6 wt.% G content to the uniform distribution of G particles and the formation of tortuous path, which hinders the diffusion of the volatile decomposition products compared to that in pure epoxy. The addition of G filler of different sizes (G150/G75 or G150/G44) had a slight difference in the values of the degraded weight and the maximum decomposition temperatures and a better thermal stability of epoxy/G150/G75 composite compared to epoxy/G150/G44 composite. According to the DSC results shown in Figure 8, attention was given to the glass transition temperatures (T_g_) of the epoxy and its composites. It was observed from the DSC curves that there was a slight deviation of T_g_ from 66 °C for epoxy/G150/G44 to 69 °C for epoxy/G150/G75 composites compared to that of pure epoxy (68 °C). Sun et al. [57] showed that the nano-sized fillers in epoxy composites had a clear effect on the reduction of T_g_ compared to composites having micro-sized fillers. They also indicated that the surface conditions of the fillers and their dispersion besides the interaction at the filler–resin interface play a crucial role for determining the T_g_ of the composites. Morimune-Moriya et al. [58] found that the lower aspect ratio filler with the higher specific surface area has better interfacial interactions between the polymer matrix and the filler, which restricted the molecular mobility of the polymer and resulted in a higher T_g_ value. In this study, using a narrow range of particle sizes of the secondary filler (i.e., 75 µm and 44 µm) might not display much difference on T_g_.

## 4. Conclusions

An experimental study to improve the electrical-mechanical behavior of epoxy/G composites based on the effects of G particle size and curing conditions was carried out, and the findings could be summarized as follows: the incorporation of multiple filler sizes of the same type increased the in-plane electrical conductivity of the composites by forming bridges of small G particles between the larger plate-like G particles, increasing the particle-to-particle contact, and consequently improving the electrical conductivity. The curing conditions temperature and time had a significant effect on increasing the electrical conductivity and tensile strength of the epoxy/G150/G75 and epoxy/G150/G44 composites. As a comparison and by optimizing the curing conditions, an increase of about 78% in the in-plane electrical conductivity of epoxy/G150 composites was achieved when combining G150 with a smaller G size compared to that containing only 80 wt.% G150, as reported in the previous work of Suherman et al. [23], and the increase in tensile strength was about 35%. Due to the poor adhesion between the filler and the epoxy and the agglomeration of the filler particles, further work is required to improve the homogeneity of the structure and optimize the curing pressure as well. There was not much difference in the transition temperature of the composites and a wider range of particle size of the secondary filler in micro- and nano-scale is recommended to be used and compared. Finally, it is also recommended to investigate in the future the optimal size of conductive fillers and the particle size gradation based on the closest accumulating theory and the particle size gradation theory.

## Figures and Tables

**Table 1:** Composition of different epoxy/G composites.

Epoxy/G Type	G150 (wt.%)	G75 (wt.%)	G44 (wt.%)	Epoxy (wt.%)
Epoxy/G150	65	0	0	35
	70	0	0	30
	75	0	0	25
	80	0	0	20
Epoxy/G150/G75	77.5	2.5	0	20
	75	5	0	20
	72.5	7.5	0	20
	70	10	0	20
Epoxy/G150/G44	77.5	0	2.5	20
	75	0	5	20
	72.5	0	7.5	20
	70	0	10	20

**Table 2:** Maximum decomposition temperatures and weight loss of the epoxy and epoxy/G composites at different temperatures.

Type	T_max_ (°C)	Weight Loss (wt.%)		
		T_100 °C_	T_200 °C_	T_max_
Epoxy	377.99	0.15	0.47	24.0
epoxy/70%G150/10%G75	369.46	0.11	0.24	11.84
epoxy/70%G150/10%G44	368.09	0.12	0.33	14.01

## Data Availability

Not applicable.

## References

[1] Chen H., Xia X.H., Yang L., He Y., Liu H. (2016). Preparation and characterization of graphite/resin composite bipolar plates for polymer electrolyte membrane fuel cells. Sci. Eng. Compos. Mater..

[2] Kakati B.K., Sathiyamoothy D., Verma A. (2010). Electrochemical and mechanical behaviour of carbon composite bipolar plate for fuel cell. Int. J. Hydrogen Energy.

[3] Thongruang W., Ritthichaiwong C., Bunnaul P., Smithmaitrie P., Chetpattananondh K. (2008). Electrical and mechanical properties of ternary composites from natural rubber and conductive fillers. Songklanakarin J. Sci. Technol..

[4] Bai X., Zhang C., Zeng X., Ren L., Sun R., Xu J. (2021). Recent progress in thermally conductive polymer/boron nitride composites by constructing three-dimensional networks. Compos. Commun..

[5] Jiang F., Zhou S., Xu T., Song N., Ding P. (2021). Enhanced thermal conductive and mechanical properties of thermoresponsive polymeric composites: Influence of 3D interconnected boron nitride network supported by polyurethane@polydopamine skeleton. Compos. Sci. Technol..

[6] Jiang F., Cui S., Rungnim C., Song N., Shi L., Ding P. (2019). Control of a dual-cross-linked boron nitride framework and the optimized design of the thermal conductive network for its thermo responsive polymeric composites. Chem. Mater..

[7] Nishata R.R.R., Sulong A.B., Sahari J., Suherman H. (2013). Effect of acid- and ultraviolet/ozonolysis-treated MW on the electrical and mechanical properties of epoxy nanocomposites as bipolar plate applications. J. Nanomater..

[8] Xiaolong L., Sheng X., Guo Y., Lu X., Wu H., Chen Y., Zhang L., Gu J. (2021). Multifunctional HDPE/CNTs/PW composite phase change material with excellent thermal and electrical conductivities. J. Mater. Sci. Technol..

[9] Suherman H., Sulong A.B., Zakaria M.Y., Nishata R.R.R., Sahari J. (2018). Electrical conductivity and physical changes of functionalized carbon nanotubes/graphite/staniless steel (SS316L)/polyprophelene composites immersed in an acidic solution. Songklanakarin J. Sci. Technol..

[10] Radzuan N.A.M., Zakaria M.Y., Sulong A.B., Sahari J. (2017). The effect of milled carbon fibre filler on electrical conductivity in highly conductive polymer composites. Compos. Part B Eng..

[11] Wang S., Huang Y., Chang E., Zhao C., Ameli A., Naguib H.E., Park C.B. (2021). Evaluation and modeling of electrical conductivity in conductive polymer nanocomposites foams with multiwalled carbon nanotube networks. Chem. Eng. J..

[12] Dweiri R. (2012). Effect of inhomogeneous size and shape of graphite particles on the in-plane electrical conductivity of PP/G/CB composites. Int. J. Mater. Res..

[13] Suherman H., Sulong A.B., Sahari J. (2010). Effect of filler loading concentration, curing temperature and molding pressure on the electrical conductivity of CNTs/graphite/epoxy nanocomposites at high loading of conductive fillers. Int. J. Mech. Mater. Eng..

[14] Heo S.I., Yun J.C., Oh K.S., Han K.S. (2006). Influence of particle size and shape on electrical and mechanical properties of graphite reinforced conductive polymer composites for the bipolar plate of pem fuel cell. Adv. Compos. Mater..

[15] Antunes R.A., Mara C.L., Oliveira D., Gerhard E., Volkmar E. (2011). Carbon materials in composite bipolar plates for polymer electrolyte membrane fuel cells: A review of the main challenges to improve electrical performance. J. Power Sources.

[16] Hui C., Hong-Bo L., Li Y., Jian-Xin L. (2010). Study on the preparation and properties of novolac epoxy/graphite composite bipolar plate for PEMFC. Int. J. Hydrogen Energy.

[17] Bairan A., Selamat M.Z., Sahadan S.N., Malingam S.D., Mohamad N. (2016). 2016. Effect of carbon nanotubes loading in multifiller polymer composite as bipolar plate for PEM fuel cell. Procedia Chem..

[18] Dweiri R., Sahari J. (2007). Electrical properties of carbon-based polypropylene composites for bipolar plates in polymer electrolyte membrane fuel cell (PEMFC). J. Power Sources.

[19] Cunningham B.D., Huang J., Baird G.B. (2007). Development of Bipolar Plates for Fuel Cells from Graphite Filled Wet-Lay Materials and a Thermoplastic Laminate Skin Layer. J. Power Sources.

[20] Hwang I.U., Yu H.N., Kim S.S., Lee D.G., Suh J.D., Lee S.H., Ahn B.K., Kim S.K., Lim T.W. (2008). Bipolar Plate Made of Carbon Fiber Epoxy Composite for Polymer Electrolyte Membrane Fuel Cells. J. Power Sources.

[21] Ma P.C., Liu M.Y., Zhang H., Wang S.Q., Wang R., Wang K., Wong Y.K., Tang B.Z., Hong S.H., Paik K.W. (2009). Enhanced Electrical Conductivity of Nanocomposites Containing Hybrid Fillers of Carbon Nanotubes and Carbon Black. ACS Appl. Mater. Interface Am. Chem. Soc..

[22] Suherman H., Sulong A.B., Sahari J. (2013). Effect of the compression molding parameters on the in-plane and through-plane conductivity of carbon nanotubes/graphite/epoxy nanocomposites as bipolar plate material for a polymer electrolyte membrane fuel cell. Ceram. Int..

[23] Suherman H., Mahyoedin Y., Septe E., Rizade R. (2019). Properties of graphite/epoxy composites: The in-plane conductivity, tensile strength and Shore hardness. AIMS Mater. Sci..

[24] Dweiri R., Suherman H., Sulong A.B., Al-Sharab J.F. (2018). Structure-property-processing investigation of electrically conductive polypropylene nanocomposites. Sci. Eng. Compos. Mater..

[25] Suherman H., Dweiri R., Mahyoedin Y., Duskiardi D. (2019). Investigation of electrical-mechanical performance of epoxy-based nanocomposites filled with hybrid electrically conductive fillers. Mater. Res. Express.

[26] Chunhui S., Mu P., Runzhang Y. (2008). The effect of particle size gradation of conductive fillers on the conductivity and the flexural strength of composite bipolar plate. Int. J. Hydrogen Energy.

[27] Dhakate S.R., Mathur R.B., Sharma S., Borah M., Dhami T.L. (2009). Influence of expanded graphite particle size on the properties of composite bipolar plates for fuel cell application. Energy Fuels.

[28] Derieth T., Bandlamudi G., Beckhaus P., Kreuz C., Mahlendorf F., Heinzel A. (2008). Development of highly filled graphite compounds as bipolar plate materials for low and high temperature pem fuel cells. J. New Mater. Electrochem. Syst..

[29] Suherman H., Duskiardi D., Suardi A., Irmayani I. (2019). Enhance the electrical conductivity and tensile strength of conductive polymer composites using hybrid conductive filler. Songklanakarin J. Sci. Technol..

[30] Dweiri R., Sahari J., Mousa A. (2010). Optimization of electrical conductivity for composite bipolar plates in PEM fuel cell. AIP Conf. Proc..

[31] Wakabayashi K., Pierre C., Dikin D.A., Ruof R.S., Ramanathan T., Brinson L.C., Torkelson J.M. (2008). Polymer-graphite nanocomposites: Efective dispersion and major property enhancement via solid-state shear pulverization. Macromolecules.

[32] Mohammadsalih Z.G., Inkson B.J., Chen B. (2020). The effect of dispersion condition on the structure and properties of polystyrene/graphene oxide nanocomposites. Polym. Compos..

[33] Ramanujam B.T.S., Radhakrishnan S. (2015). Solution-blended polyethersulfone-graphite hybrid composites: Formation of nanographite and electrical characterization. J. Thermoplast. Compos. Mater..

[34] Hu N., Masuda Z., Yamamoto G., Fukunaga H., Hashida T., Qiu J. (2008). Effect of fabrication process on electrical properties of polymer/MWCNTs nanocomposite. Compos. Part A.

[35] Chandrasekaran S., Seidel C., Schulte K. (2013). Preparation and characterization of graphite nano-platelet (GNP)/epoxy nanocomposite: Mechanical, electrical and thermal properties. Eur. Polym. J..

[36] Li M.D., Niu H., Yang H. (2014). Effects of the preparation parameters on the properties of graphite/carbon fiber/copper/phenolic resin composites. Appl. Mech. Mater..

[37] Zheng G., Wu J., Wang W., Pan C. (2004). Characterizations of expanded graphite/polymer composites prepared by in situ polymerization. Carbon.

[38] Calixto C.M.F., Mendes R.K., Oliveira A.C., Ramos L.A., Cervini P., Cavalheiro É.T.G. (2007). Development of graphite-polymer composites as electrode materials. Mater. Res..

[39] Naz A., Kausar A., Siddiq M. (2016). Influence of graphite filler on physicochemical characteristics of polymer/graphite composites: A review. Polym.-Plast. Technol. Eng..

[40] Alshammari B.A., Al-Mubaddel F.S., Karim M.R., Hossain M., Al-Mutairi A.S., Wilkinson A.N. (2019). Addition of graphite filler to enhance electrical, morphological, thermal, and mechanical properties in poly (ethylene terephthalate): Experimental characterization and material modeling. Polymers.

[41] Mokhtari M., Archer E., Bloomfield N., Harkin-Jones E., Mcilhagger A. (2021). Melt-blended multifunctional PEEK/expanded graphite composites. Front. Mater..

[42] Alo O., Otunniyi I.O. (2021). Electrical conductivity of polyethylene/epoxy/graphite/carbon black composites: Synergy of blend immiscibility and hybrid filler. Polym.-Plast. Technol. Mater..

[43] Ramanujam B.T.S., Annamalai P.K., Thakur V.K., Thakur M.K., Pappu A. (2017). 1-Conducting polymer-graphite binary and hybrid composites: Structure, properties, and applications. Hybrid Polymer Composite Materials.

[44] Sengupta R., Bhattacharya M., Bandyopadhyay S., Bhowmick A.K. (2011). A review on the mechanical and electrical properties of graphite and modified graphite reinforced polymer composites. Prog. Polym. Sci..

[45] Yue L., Pircheraghi G., Monemian S.A., Manas-Zloczower I. (2014). Epoxy composites with carbon nanotubes and graphene nanoplatelets-dispersion and synergy effects. Carbon.

[46] Ghaleb Z., Mariatti M., Ariff Z. (2017). Synergy Effects of graphene and multiwalled carbon nanotubes hybrid system on properties of epoxy nanocomposites. J. Reinf. Plast. Compos..

[47] Radzuan N.A.M., Sulong A.B., Iswandi I. (2021). Effect of multi-sized graphite filler on the mechanical properties and electrical conductivity. Sains Malays..

[48] Szeluga U., Pusz S., Kumanek B., Olszowska K., Kobyliukh A., Trzebicka B. (2021). Effect of graphene filler structure on electrical, thermal, mechanical, and fire retardant properties of epoxy-graphene nanocomposites-a review. Crit. Rev. Solid State Mater. Sci..

[49] Clingerman M.L., Weber E.H., King J.A., Schulz K.H. (2003). 2003. Development of an Additive Equation for Predicting the Electrical Conductivity of Carbon-Filled Composites. J. Appl. Polym. Sci..

[50] Kara S., Arda E., Dolastir F., Pekcan Ö. (2010). Electrical and optical percolations of polystyrene latex–multiwalled carbon nanotube composites. J. Colloid Interface Sci..

[51] Causin V., Marega C., Marigo A., Ferrara G., Ferraro A. (2006). Morphological and structural characterization of polypropylene/conductive graphite nanocomposites. Eur. Polym. J..

[52] Mathur R.B., Dhakate S.R., Gupta D.K., Dhami T.L., Aggarwal R.K. (2008). Effect of different carbon fillers on the properties of graphite composite bipolar plate. J. Mater. Process. Technol..

[53] Martin C.A., Sandler J.K.W., Shaffer M.S.P., Schwarz M.K., Bauhofer W., Schulte K., Windle A.H. (2004). Formation of Percolating Networks in Multi-Wall Carbon-Nanotube–Epoxy Composites. Compos. Sci. Technol..

[54] Bera T., Acharya S.K., Mishra P. (2018). Synthesis, mechanical and thermal properties of carbon black/epoxy composites. Int. J. Eng. Sci. Technol..

[55] Mochane M.J., Motaung T.E., Motloung S.V. (2017). Morphology, flammability, and properties of Graphite Reinforced Polymer Composites. Syst. Rev. Polym. Compos..

[56] Bhagat S. (2013). Analysis of thermal behavior of graphite flakes filled epoxy composites. Indian J. Appl. Res..

[57] Sun Y., Zhang Z., Moon K.S., Wong C.P. (2004). Glass transition and relaxation behavior of epoxy nanocomposites. J. Polym. Sci. Part B Polym. Phys..

[58] Morimune-Moriya S., Goto T., Nishino T. (2019). Effect of aspect ratio of graphene oxide on properties of poly (vinyl alcohol) nanocomposites. Nanocomposites.

